# Eight Hours a Day

**Published:** 1879-10

**Authors:** 


### EIGHT HOURS A DAY.

There can be no doubt that growing persons are often overworked, especially boys on a farm from twelve to eighteen; the danger is increased in proportion to the rapidity of the growth. Persons who work hard. under twenty years of age, should be allowed ten hours rest in bed. The health of girls is sometimes ruined by over pushing mothers. The desire of some constitutions to remain in bed awhile after waking up is inappeasible, and to have to get up is literally dreadful, and involves an amount of self-denial and sacrifice almost inconceivable. There is no merit whatever in simple early rising and a great deal of nonsense has been written on the subject; it is always a cruelty and a crime to the young, and to a great extent to all unless it be preceeded by an early retiring. One of the most criminal of robberies is that which abridges the hours of necessary sleep. But as to the working only eight hours a day for grown people, the great mass of mechanics, it is the vagary of an impracticable noodle. If a man working by the job can earn as much money as will support him, and is satisfied with that, let him do it, but to demand for the labor of eight hours the compensation due to ten or twelve hours, is a theft in disguise. Once establish such a principle, and before we are aware of it we would find the poor, the shiftless and improvident demanding of the frugal and care-taking classes five dollars a day for the trouble of spending it. This banding together of one class of men to compel them to give them what they ask for their commodities, whether labor, time or goods, is that excess of democracy, which if anything will, will destroy this government, and one of two things only can prevent it, either an educated religious sentiment or a property qualification to the native born only, as voters.

There is one thing in this connection highly discreditable to a class of persons of whom we have a right to expect better things. Printers in this city and some of the editors, contributed their money and influence, last winter, towards encouraging men to demand more money for their labor than that labor was worth. These men not only refused to work themselves, but by threats and actual violence prevented others from working who would gladly have accepted the vacated positions. Nothing in a long time has so signally developed the worthlessness of the city press as a guide for what is legal, just and right. Some of the papers took openly the side of the strikers, others in more covert ways, threw their influence in the wrong direction. The fact is the leading newspapers of large cities know but seven principles, the five loaves and the two fishes; whatever side promises the most gain, that side for the time being they advocate, and that leads to a question of very extensive application, whether it is not the duty of the friends of religion throughout the country to patronize the secular press less

There are but one or two secular papers in the whole city of New-York whose abiding, fundamental influence is not against evangelical religion; favorable to it generally as far as the expression of sentiment is concerned, but greedy always of facts which can be used against the clergy, the Bible and the Sabbath day.

For any man in a free country to dictate to his employer how much he shall pay a workman for a day's work and then turn round and dictate how many hours shall constitute that day's work argues an impudence, an effrontery, and a despotism not permissable in any aristocracy on earth; and yet the chief papers in New-York city, in a manner more or less direct, have countenanced this same thing.

As to the practical effect in these disjointed days of less time and more money for workmen, men all over the city who employ many hands say, and the mechanics admit workmen have less money now than in the good old times before the war; while work is not done so well, and the number of good workmen is steadily decreasing, because making so much more money they take more holidays during which the money is squandered in drinking, carousing and various extravagances, while the really good men who still work hard and save are tempted by the unusual amount of money they handle to embark in more remunerative forms of business, this makes hands scarce and brings them in brisk demand; so much is this the case that mechanics have got to believe they are doing employers a favor to work for them: this generates a feeling of independence, and then follows a carelessness in work and an insubordination expressing itself often in words. "I can get work and wages elsewhere," and off they go.

The truth is a day's work should be measured "from the rising of the sun to the going down of the same," at least in in our latitudes, whether it be winter or summer; one good result would certainly flow from it during a larger portion of the year: when night came the workman would be glad enough to go to bed, instead of wandering around to drinking houses, billiard rooms, theatres, dance houses, and waiter-girl saloons.

